# Effects of combination treatment with alendronate and raloxifene on skeletal properties in a beagle dog model

**DOI:** 10.1371/journal.pone.0181750

**Published:** 2017-08-09

**Authors:** Matthew R. Allen, Erin McNerny, Mohammad Aref, Jason M. Organ, Christopher L. Newman, Brian McGowan, Tim Jang, David B. Burr, Drew M. Brown, Max Hammond, Paul R. Territo, Chen Lin, Scott Persohn, Lei Jiang, Amanda A. Riley, Brian P. McCarthy, Gary D. Hutchins, Joseph M. Wallace

**Affiliations:** 1 Department of Anatomy and Cell Biology, Indiana University School of Medicine, Indianapolis, Indiana, United States of America; 2 Department of Orthopedics, Indiana University School of Medicine, Indianapolis, Indiana, United States of America; 3 Department of Biomedical Engineering, Indiana University Purdue University of Indianapolis, Indianapolis, Indiana, United States of America; 4 Department of Radiology and Imaging Sciences Indiana University School of Medicine, Indianapolis, Indiana, United States of America; Rensselaer Polytechnic Institute, UNITED STATES

## Abstract

A growing number of studies have investigated combination treatment as an approach to treat bone disease. The goal of this study was to investigate the combination of alendronate and raloxifene with a particular focus on mechanical properties. To achieve this goal we utilized a large animal model, the beagle dog, used previously by our laboratory to study both alendronate and raloxifene monotherapies. Forty-eight skeletally mature female beagles (1–2 years old) received daily oral treatment: saline vehicle (VEH), alendronate (ALN), raloxifene (RAL) or both ALN and RAL. After 6 and 12 months of treatment, all animals underwent assessment of bone material properties using *in vivo* reference point indentation (RPI) and skeletal hydration using ultra-short echo magnetic resonance imaging (UTE-MRI). End point measures include imaging, histomorphometry, and mechanical properties. Bone formation rate was significantly lower in iliac crest trabecular bone of animals treated with ALN (-71%) and ALN+RAL (-81%) compared to VEH. In vivo assessment of properties by RPI yielded minimal differences between groups while UTE-MRI showed a RAL and RAL+ALN treatment regimens resulted in significantly higher bound water compared to VEH (+23 and +18%, respectively). There was no significant difference among groups for DXA- or CT-based measures lumbar vertebra, or femoral diaphysis. Ribs of RAL-treated animals were smaller and less dense compared to VEH and although mechanical properties were lower the material-level properties were equivalent to normal. In conclusion, we present a suite of data in a beagle dog model treated for one year with clinically-relevant doses of alendronate and raloxifene monotherapies or combination treatment with both agents. Despite the expected effects on bone remodeling, our study did not find the expected benefit of ALN to BMD or structural mechanical properties, and thus the viability of the combination therapy remains unclear.

## Introduction

The skeletal biology community has declared a crisis in osteoporosis treatment [1]. The use of bisphosphonates, the longtime gold-standard treatment for bone loss, has declined due to concerns over rare, but serious side effects such as osteonecrosis of the jaw and atypical femoral fractures [2,3]. Although other anti-remodeling agents such as denosumab and raloxifene and anabolic agents such as tereparatide are approved for use and effective [4], there is a clear need to develop newer strategies for long-term preservation of skeletal health.

A growing number of studies have investigated combination treatment as an approach to treat bone disease [5]. These studies, both clinical and preclinical, have taken various approaches (co-administration or sequential treatment) and most often have combined anti-resorptive and anabolic treatments due to their distinctly different mechanisms of action. Recent data have documented that raloxifene, an anti-resorptive, has positive effects on the mechanical properties. Specifically, raloxifene can act through non-cellular pathways (effects exists in devitalized bone) to modify the hydration of the bone matrix and thus enhanced the mechanical properties (specifically to toughness) [6]. These effects of raloxifene have been shown, both *in vitro* and *in vivo* [6–10], to reverse the bisphosphonate-induced reduction of tissue-level bone toughness [11]. The lone clinical study examining combination of alendronate and raloxifene showed modest effects relative to either monotherapy, but outcomes were restricted to serum biomarkers of bone formation/resorption and bone mineral density (BMD), and bone mechanical properties were not evaluated [12].

The goal of this study was to investigate the combination of alendronate and raloxifene with a particular focus on mechanical properties. To achieve this goal we utilized a large animal model, the beagle dog, used previously by our laboratory to study both alendronate and raloxifene monotherapies. In these past studies, we have shown that clinically-relevant doses of alendronate have a significant positive effect on bone mass, bone remodeling suppression, and bone ultimate load while also documenting a significant reduction in tissue toughness [13–21]. Conversely we have documented that monotherapy with raloxifene has only modest effects on bone mass and remodeling suppression, but also significant positive effects on ultimate load and toughness, the latter being associated with improvements in tissue hydration [7,22,23]. It is based on the scientific premise of these previous datasets that the current study was designed. We hypothesized that combining alendronate and raloxifene treatment would improve bone’s mechanical properties more than either drug alone by allowing the alendronate-induced increases in bone density (volume and mineralization) and the raloxifene-induced benefits to bone material properties.

## Methods

### Animals and study design

All procedures were approved by the Indiana University School of Medicine IACUC prior to initiating the study. Forty-eight skeletally mature female beagles (1.2 ± 0.1 years old) were purchased from Marshall Farms USA (North Rose, NY). Following two weeks of acclimatization animals began daily oral treatment: saline vehicle (VEH; 1 ml/kg/day), alendronate (ALN, 0.2 mg/kg/day), raloxifene (RAL, 0.5 mg/kg/day) or the combination of ALN and RAL (ALN+RAL). ALN (synthesized by the IU Clinical chemistry core) was mixed in sterile saline while raloxifene (Eli Lilly) was mixed in 10% hydroxypropyl-ß-cyclodextrin (Sigma); the latter to facilitate going into solution and to increase palatability for oral dosing. The ALN and RAL doses represent the clinical dose equivalent of daily dosing used for the treatment of post-menopausal osteoporosis and match the doses we have previously shown to have skeletal benefits in a dog model [22]. Dosing was conducted in the morning, prior to any feeding, for one year. Animals were housed in standard laboratory housing conditions and received daily husbandry care including access to outside runs when weather was appropriate.

After 6 and 12 months of treatment, all animals underwent assessment of bone material properties using *in vivo* reference point indentation; a subset of animals had in vivo assessment of skeletal hydration using ultra-short echo magnetic resonance imaging at two time points (*see details below*). To label active bone remodeling sites at the end of the experiment, animals were injected with calcein (5 mg/kg, intravenous) using a 2-12-2-5 schedule (two days of dose, twelve days off, two more days of dose, five days to euthanasia). Animals were euthanized by intravenous administration of sodium pentobarbital after 1 year of treatment. Tissues were collected and stored frozen wrapped in saline-soaked gauze (for imaging/mechanical testing) or fixed in 10% neutral buffered formalin (for histology).

### In vivo mechanical assessment

Reference point indentation [24] and Osteoprobe tests [25] were conducted as previously described in detail. Two different methods were used in order to compare them against each other and determine their respective ability to detect treatment-induced differences. Briefly, animals were sedated and local anesthesia was administered to the testing site. Reference point indentation (RPI: BioDent Hif, Active Life Scientific, Santa Barbara, CA) was conducted on the right anterior tibia midshaft using a protocol designed for clinical studies. The periosteum was scraped from the underlying cortex, a reference force of ~13 N was applied to stabilize the unit, and the measurement protocol was initiated (four preconditioning cycles at a force of 1 N and a frequency of 5 Hz followed by a series of 10 testing cycles at 10 N and 2 Hz). Five measurements, within a few mm of each other, were collected on each animal. If a test was found to be unusable during the live animal testing, a replacement was run. In cases where the data were found after the fact to be implausible (for instance a negative indentation distance that was not caught during the in vivo test), it was not used in the analysis leaving some animals with only four tests. Key outcome parameters include first cycle indentation distance, unloading slope, indentation distance increase, total indentation distance, and total energy absorption.

Osteoprobe measurements were performed on the left tibia of each dog at a location similar to RPI on the right limb. Following administration of a local anesthetic the test probe was carefully inserted through the lifted skin and positioned normal to the bone surface (based on the judgement of the individual doing the testing). The device was slowly lowered to activate the indentation cycle, which monitors the indentation depth increase resulting from an impact load of 30N superimposed on a 10 N triggering preload (40 N total force). Each Osteoprobe measurement session consisted of 5 indentations located at least 2 mm apart along a line parallel to the long axis of the diaphysis and was performed without removing the probe from the skin between indents. The direction of this spacing was switched (moving proximally or distally from the midpoint) between 6 and 12 months to avoid indenting a previously tested site. In some cases (30 out of 96 test sessions), 1–5 additional measurements were made based on our assessment that one or more indents were questionable and warranted flagging for further evaluation. Five indents on the manufacturer-provided poly(methyl methacrylate) block were performed immediately following each bone test, both to calibrate the system to each individual probe and to allow calculation of the bone material strength index (BMSi). BMSi represents the indentation depth on the bone relative to the plastic block.

All animals were conscious and mobile within 30 minutes post-testing using a modification of the Glasgow Composite Pain Scale. There was no sign of pain or discomfort based on pain scoring taken within the first 8–12 h post-test, and then again 24 h post-test thus no post-operative pain medications were given. Data analyses were conducted using methods previously described in substantial detail by our group for both Biodent [24] and Osteoprobe [25] machines.

### Ultra-short Echotime MRI (UTE-MRI)

Detailed methods for imaging and analysis have been previously described [7]. Briefly, one half of the animals in each group (n = 6/group) were anesthetized (ketamine/diazepam followed by inhalation isoflurane) and the hind limbs were immobilized in a custom configured splint that permitted precise placement of the two channel Miniflex^®^ surface coils (Rapid MR International) laterally over the diaphysis inferior to the tibial plateau, closely matching the region of in vivo mechanical assessment. Each animal was scanned on an Siemens 3T Tim Trio MRI using a 3D UTE sequence with the following characteristics: TR (Time to repeat the sequence) 20 ms; TE1 (Echo time 1) variable (0.05, 0.06, 0.07, 0.08, 0.10, 0.12, 0.14, 0.20, 0.30, 0.40, 0.50, 0.60, 0.80, 1.0, 1.1 ms); TE2 (Echo time 2) 5 ms; Fat Saturation; Average 1, Excitation Flip Angle 50°; Normalization Filter; Acquisition Matrix 80x80x80 mm; Field of View 50x50 mm; Spatial Resolution 0.63x0.63x0.63 mm, and TA (Total acquisition time) 28 min.

Image volumes for both variable (TE1) and fixed (TE2) echo times were imported, segmented, and quantified using Analyze 11.0 (AnalyzeDirect). Marrow and cortical bone for each image series per animal were segmented on the shortest TE1 image using a region growing technique, where the distal and proximal limits were prescribed at a fixed distance from the center of the field of view. Segmented regions were then extracted for all TE1 and TE2 images volumes, thereby permitting secondary analysis of the UTE signal. Receiver gain offset differences between successive images were scaled using previously published equations [7]. To improve model fits in low signal to noise data, images were corrected and individually modeled using a double exponential decay [26,27], with the minor modifications [7], to compute percent free and bound water.

### Dynamic histomorphometry

Iliac crests and ribs (9^th^ right) were processed for histology following previously published methods [14]. Following embedding in methyl methacrylate, iliac crest sections were cut using a microtome (4 μm) and cover-slipped unstained. Embedded ribs were cut using a wire-saw (80–100 μm) and mounted unstained. Rib analyses were conducted within the cortex of one section to assess intracortical remodeling rate. The number of osteons with label (Labelled osteon #), label length, and distance between labels (mineral apposition rate, MAR) were quantified and normalized to cortical bone area to calculate bone formation rate (BFR) as previously described [13]. Iliac crest samples were measured within the trabecular bone region (approximately an 8 mm^2^ region of interest) of one section for amount of single and double labelled surface (to calculate mineralizing surface/bone surface (MS/BS) and distance between labels (MAR) for calculation of bone formation rate (BFR) [14]. All nomenclature is in accordance with published standards [28].

### Ex vivo skeletal imaging

Prior to mechanical testing bones, various imaging modalities were utilized to determine BMD or bone architecture/geometry. Lumbar vertebrae (L4) were scanned using dual-energy x-ray absorptiometry (DXA) for assessment of overall areal bone mineral density (aBMD) and bone mineral content (BMC) as previously described [14]. Vertebrae and femoral necks were scanned with microCT (Skyscan 1172 and 1176, respectively) to determine bone volume/tissue volume ratios (BV/TV) along with trabecular thickness and number. Ribs (right 11^th^) and femoral diaphyses were scanned with peripheral quantitative computed tomography (pQCT) to determine bone area and cross-sectional moment of inertia. All nomenclature is in accordance with published suggestions [29].

### Ex vivo mechanical testing

Lumbar vertebrae (L4) and femoral necks were tested in compression; the femoral mid-diaphysis, and rib were tested in three-point bending. Both compression and bending tests represent quasi-static uniaxial conditions. L4 were thawed to room temperature, end plates removed (low speed saw under irrigation) and then tested at a rate of 0.5 mm/min on a MiniBionix system (MTS) [14]. Femoral three-point bending was conducted in the posterior/anterior direction using a bottom fixture span length of 50 mm centered at the mid-point with a displacement rate of 60 mm/min on a MiniBionix system (MTS). Following testing, the proximal femur was potted in low melting point alloy and the head of the femur compressed downward at 1.8 mm/min on a servohydraulic system (Test Resources). Ribs were tested at the point of greatest curvature, with a lower span length of 25 mm, at a displacement rate of 20 mm/min on a servohydraulic system (Test Resources). Load/displacement curves were analyzed using customized MATLAB codes for determination of ultimate load, stiffness, displacement, and energy absorption. For the rib and femoral diaphysis, material properties were calculated by accounting for bone geometry using standard methods [30].

### Statistics

Statistical assessment of endpoint data was conducted using a one-way ANOVA followed by post-hoc tests (protected LSD) when the F-value was significant. Outcome measures with longitudinal data (in vivo mechanics and UTE-MRI) were assessed using two-way ANOVA (drug and time) with repeated measures followed by simple main effects analysis when the F-value was significant. For all tests, p values ≤ 0.05 were used for statistical significance. Data are presented as mean and standard deviation.

## Results

There was no significant difference in animal body weight at baseline or at the end of the experiment (Table 1). Overall, all animals gained significant body weight with no difference in gain among groups. There was no difference in femoral length or width among groups at the end of the experiment (Table 1).

**Table 1:** Body mass and femoral dimensions.

	VEH	ALN	RAL	ALN + RAL	ANOVA p Value
Initial body mass, kg	7.6 ± 0.7	7.6 ± 1.3	7.4 ± 1.2	7.5 ± 0.7	0.978
Final body mass, kg	9.1 ± 0.8	8.9 ± 1.1	8.5 ± 1.3	8.4 ± 1.1	0.404
Femoral length, mm	101 ± 5	105 ± 9	104 ± 5	106 ± 8	0.372
Femoral width (M-L), mm	9.7 ± 0.5	9.7 ± 0.7	9.4 ± 0.7	9.6 ± 0.5	0.690
Femoral width (A-P), mm	8.8 ± 0.5	8.7 ± 0.6	8.7 ± 0.5	8.9 ± 0.6	0.672

Data presented as mean and standard deviation. All sample sizes = 12/group.

**Fig 1:**
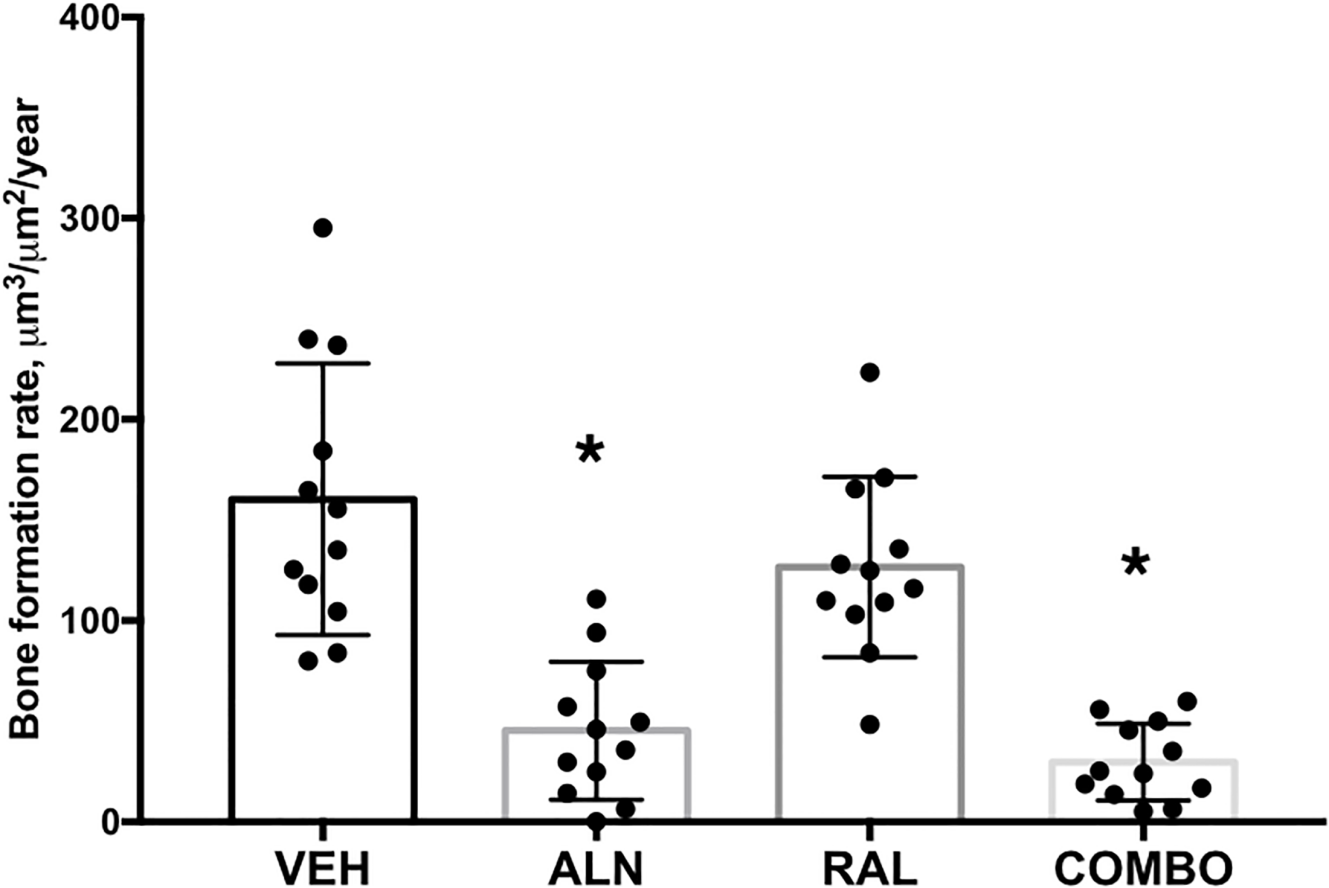
Iliac crest trabecular bone formation rate. * p < 0.05 versus vehicle.

Bone formation rate was significantly lower in iliac crest trabecular bone of animals treated with ALN (-71%) and ALN+RAL (-81%) compared to VEH (Fig 1). Trabecular MS/BS was significantly lower in all three treatment groups relative to VEH (RAL -21%; ALN -65%; ALN+RAL—80%) while MAR was lower than VEH only in ALN and ALN+RAL groups (Table 2). Intracortical bone formation rate of the rib was not significantly different than VEH in any of the three treatment groups (Table 2).

**Table 2:** Dynamic histomorphometry of trabecular and cortical bone.

	VEH	ALN	RAL	ALN + RAL	ANOVA p value
**Iliac crest**					
Animals with double label, #	12	11	12	12	
Mineral apposition rate, μm/day	1.28 ± 0.20	0.94 ± 0.37 *^	1.29 ± 0.29	1.04 ± 0.22 *^	**0.006**
Mineralizing surface/Bone surface, %	33.8 ± 11.3	12.0 ± 8 *^	26.5 ± 5.7 *	7.44 ± 4.1 *^	**0.0001**
**Rib**					
Animals with double label, #	11	11	11	11	
Mineral apposition rate, μm/day	1.06 ± 0.39	1.07 ± 0.39	1.09 ± 0.4	0.99 ± 0.52	0.995
Labelled osteons, #/mm^2^	3.2 ± 2.7	1.8 ± 1.4	1.82 ± 2.02	0.95 ± 0.77 *	**0.044**
Intracortical bone formation rate, %/year	17.9 ± 16.7	9.7 ± 9.1	11.3 ± 10.9	6.4 ± 5.7	0.105

Data presented as mean and standard deviation. All sample sizes = 12/group. p < 0.05 vs (*) VEH, (^) RAL

**Fig 2:**
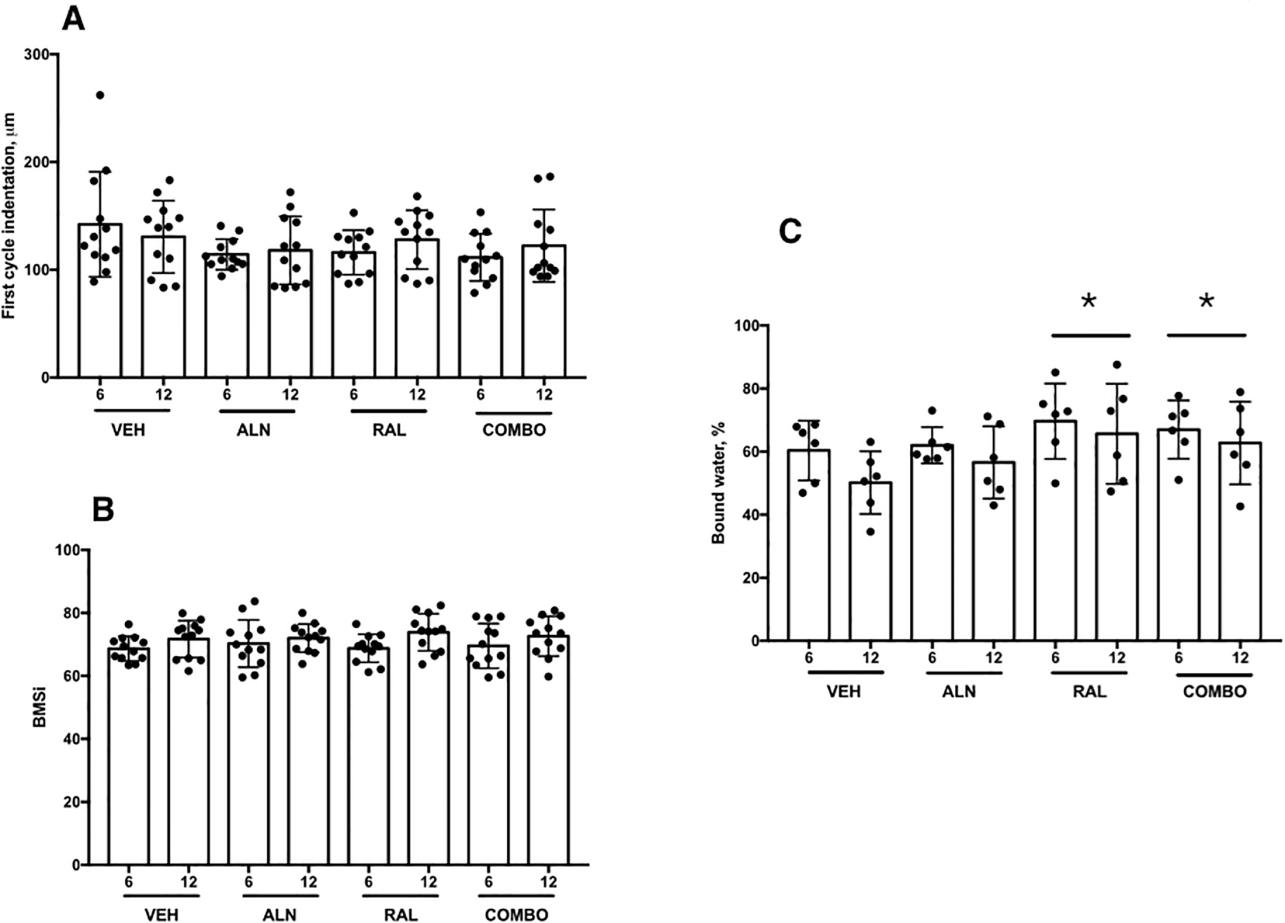
In vivo assessments after 6 and 12 months of treatments among the groups. (A) First cycle indentation depth from BioDent cyclic indentation device showed significant group effects but no effect of time or interaction between group and time. (B) Bone material strength index (BMSi) from Osteoprobe impact testing showed no significant effect of treatment or time. (C) Bound water from UTE-MRI measures of the proximal tibia showing higher percentages in raloxifene and combination treatments relative to vehicles. Gray bars represent 6 months; Black bars represent 12 months. * p < 0.05 when pooled across groups relative to VEH group.

In vivo assessment of the tibia using BioDent RPI revealed no significant effects of time, treatment, or interaction between for any variable (Table 3, Fig 2). There was a significant main effect of time (but not treatment or interaction) for BMSi with values at 12 months higher than those at 6 months (Fig 2). There was a significant main effect of treatment on bound/free water of the proximal tibia with RAL and RAL+ALN treatment regimens; both resulted in significantly higher bound water compared to VEH (+23 and +18%, respectively; Fig 2).

**Table 3:** In vivo tibia indentation and hydration properties after 6 and 12 months of treatment.

	Time point	VEH	ALN	RAL	ALN + RAL	Drug effect	Time effect	Interaction
**BioDent**								
First cycle indentation distance (ID), μm	6 mth	142 ± 49	114 ± 14	116 ± 21	111 ± 22	0.054	0.581	0.591
	12 mth	131 ± 36	118 ± 32	128 ± 27	122 ± 34			
Unloading slope, N/mm	6 mth	0.43 ± 0.06	0.43 ± 0.06	0.43 ± 0.04	0.46 ± 0.06	0.668	0.452	0.263
	12 mth	0.45 ± 0.07	0.46 ± 0.06	0.43 ± 0.06	0.44 ± 0.03			
Indentation distance increase (IDI), μm	6 mth	13.0 ± 1.5	12.6 ± 1.2	12.0 ± 1.6	11.5 ± 1.8	0.124	0.609	0.218
	12 mth	13.2 ± 2.0	11.9 ± 1.6	12.1 ± 1.5	12.6 ± 1.7			
Total ID, μm	6 mth	149 ± 50	121 ± 15	122 ± 21	117 ± 22	0.053	0.569	0.604
	12 mth	137 ± 34	124 ± 32	134 ± 28	239 ± 34			
Total energy, μJ	6 mth	912 ± 131	859 ± 108	869 ± 138	897 ± 135	0.353	0.053	0.864
	12 mth	1009 ± 230	890 ± 175	957 ± 200	941 ± 145			
**Osteoprobe**								
Bone material strength index (BMSi)	6 mth	68.7 ± 3.9	70.3 ± 7.5	68.8 ± 4.5	69.6 ± 7.1	0.935	**0.003**	0.717
	12 mth	71.7 ± 5.9	72.0 ± 4.5	73.8 ± 5.9	72.6 ± 6.3			
**UTE-MRI**								
Bound water, %	6 mth	60.4 ± 9.5	62.0 ± 5.8	69.7 ± 12.0	67.0 ± 9.3	**0.050** #	0.087	0.901
	12 mth	50.2 ± 9.9	56.6 ± 11.5	65.7 ± 15.9	62.7 ± 13.1			
Free water, %	6 mth	39.6 ± 9.5	38.0 ± 5.8	30.3 ± 12.0	33.0 ± 9.3	**0.050** #	0.087	0.901
	12 mth	49.8 ± 9.9	43.4 ± 11.5	34.3 ± 15.9	37.3 ± 13.1			

Data presented as mean and standard deviation. Sample sizes for RPI and Osteoprobe measures = 12/group; sample size for UTE-MRI = 6/group.

^#^ significant main effect of RAL and RAL+ALN versus VEH when values across times are pooled.

There was no significant difference among groups for DXA-based measures of the entire 4^th^ lumbar vertebra body or micro-CT based measures of trabecular bone (Table 4). Composite (both cortical and trabecular bone together) analysis of the central femoral neck revealed no significant effect of any treatment on total bone volume or BV/TV (Table 4). There was no significant effect of treatment on femoral mid-diaphysis cortical bone density or geometry (Table 4). Cortical BMC, BMD and area of the rib were all significantly affected by drug treatment (Table 4). Cortical BMC and cortical area of RAL-treated animals was significantly lower than all other groups while cortical BMD was significantly higher in ALN+RAL compared to all other groups.

**Table 4:** Bone density, architecture and geometry.

	VEH	ALN	RAL	ALN + RAL	ANOVA p value
**Vertebra**					
Areal bone mineral density (BMD), mg/mm^2^	0.87 ± 0.11	0.88 ± 0.09	0.85 ± 0.13	0.86 ± 0.07	0.859
Bone mineral content (BMC), mg/mm	1.18 ± 0.25	1.25 ± 0.14	1.13 ± 0.18	1.18 ± 0.18	0.482
Bone area, mm^2^	1.36 ± 0.23	1.42 ± 0.14	1.35 ± 0.18	1.38 ± 0.16	0.776
Bone volume / tissue volume (BV/TV), %	21.6 ± 3.6	21.8 ± 4.7	20.8 ± 3.1	20.6 ± 3.0	0.799
Cross-sectional tissue area, mm^2^	137 ± 14	145 ± 20	135 ± 16	145 ± 17	0.324
Trabecular number, #/mm^2^	2.1 ± 0.3	2.1 ± 0.3	2.1 ± 0.2	2.1 ± 0.3	0.877
Trabecular thickness, μm	102 ± 7	104 ± 11	100 ± 10	95 ± 6	0.149
**Femoral Neck**					
Total volume, mm^2^	84 ± 32	83 ± 45	84 ± 32	86 ± 25	0.996
Bone volume, mm^2^	39 ± 16	37 ± 23	42 ± 22	37 ± 14	0.940
BV/TV, %	46 ± 10	45 ± 14	49 ± 14	44 ± 14	0.751
**Femoral Diaphysis**					
Bone area, mm^2^	44.7 ± 3.7	45.3 ± 4.9	44.6 ± 4.1	45.8 ± 4.8	0.903
Cortical thickness, mm	1.89 ± 0.17	1.95 ± 0.16	1.97 ± 0.13	2.00 ± 0.18	0.469
Cross-sectional moment of inertia, mm^4^	314 ± 74	305 ± 77	289 ± 75	311 ± 77	0.866
**Rib**					
Cortical area, mm^2^	6.49 ± 0.56	6.60 ± 0.73	5.65 ± 0.88 *#&	6.68 ± 0.90	**0.008**
Cortical BMC, mg/mm	7.33 ± 0.67	7.50 ± 0.80	6.41 ± 1.06 *#&	7.74 ± 1.05	**0.005**
Cortical BMD, mg/mm^2^	1128 ± 24	1138 ± 31	1133 ± 24	1160 ± 21 *#^	**0.023**
Cross-sectional moment of inertia, mm^4^	7.53 ± 1.6	7.24 ± 1.7	5.73 ± 1.7	7.41 ± 2.18	0.066

Data presented as mean and standard deviation. All sample sizes = 12/group. p < 0.05 vs (*) VEH, (#) ALN, (^) RAL, (&) Combo

**Fig 3:**
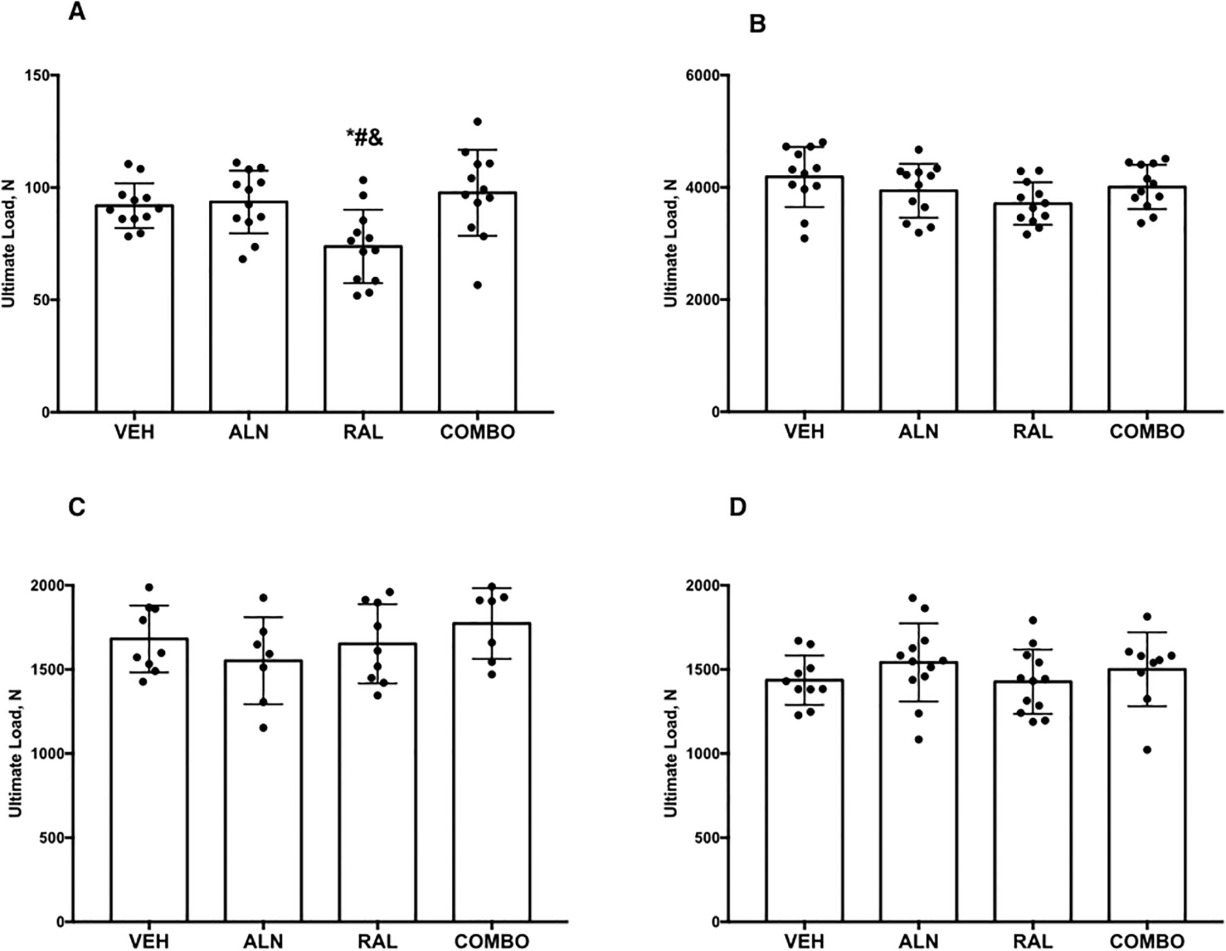
Whole bone ultimate load from the (A) rib, (B) vertebrae, (C) femoral neck and (D) femoral diaphysis. p < 0.05 vs (*) VEH, (#) ALN, (&) Combo.

There was a significant effect of treatment on multiple structural mechanical properties of the rib (Table 5, Fig 3). Animals treated with RAL had significantly lower ultimate load and work to failure relative to VEH and lower stiffness relative to ALN+RAL There were no significant differences among groups for estimated material properties. There were no differences in structural or estimated material mechanical properties of the femoral diaphysis or femoral neck (Table 5). Interestingly, during the femoral neck tests, 6 of the 12 bones from animals treated with ALN+RAL failed in the shaft region as opposed to the neck. No other treatment group had any bones fracture at the shaft.

**Table 5:** Structural and estimated material properties.

	VEH	ALN	RAL	ALN + RAL	ANOVA p value
**Rib**	12	12	12	12	
Ultimate load, N	92 ± 10	94 ± 14	74 ± 16 *#&	98 ± 19	**0.002**
Stiffness, N/mm	119 ± 17	124 ± 24	103 ± 21 #&	132 ± 28	**0.028**
Total displacement, μm	5664 ± 951	5055 ± 1000	5667 ± 806	4927 ± 626	0.071
Energy to failure, Nmm	416 ± 57	397 ± 100	328 ± 68 *#	393 ± 79	**0.049**
Ultimate stress, MPa	204 ± 29	212 ± 41	187 ± 44	221 ± 27	0.139
Modulus, GPa	6.6 ± 0.9	7.4 ± 1.4	7.6 ± 0.9	7.8 ± 1.1	0.067
Toughness, MJ/m^3^	36.9 ± 8.3	33.7 ± 7.7	29.9 ± 9.7	34.1 ± 4.3	0.193
**Vertebra**	12	12	12	12	
Ultimate load, N	4187 ± 536	3940 ± 480	3711 ± 379	4008 ± 394	0.093
Stiffness, N/mm	12193 ± 4449	11791 ± 3381	9833 ± 2006	12301 ± 3163	0.253
Displacement to ultimate load, μm	666 ± 182	641 ± 218	696 ± 200	599 ± 197	0.685
Energy to ultimate load, Nmm	1647 ± 483	1352 ± 481	1333 ± 365	1336 ± 119	0.236
**Femoral neck**	9	7	9	6	
Ultimate load, N	1681 ± 199	1552 ± 259	1653 ± 235	1773 ± 210	0.347
Stiffness, N/mm	709 ± 159	683 ± 151	675 ± 89	617 ± 130	0.596
Displacement to Ultimate load, μm	3339 ± 366	4051 ± 770	4246 ± 1023	3921 ± 836	0.105
Energy to Ultimate load, Nmm	3057 ± 388	3564 ± 1127	3969 ± 1444	3575 ± 847	0.335
**Femoral diaphysis**	10	12	12	10	
Ultimate load, N	1436 ± 147	1542 ± 233	1427 ± 191	1501 ± 220	0.479
Stiffness, N/mm	1276 ± 157	1410 ± 221	1334 ± 197	1369 ± 271	0.521
Total displacement, μm	2879 ± 340	3027 ± 356	2802 ± 582	3029 ± 223	0.641
Energy to failure, Nmm	3152 ± 424	3623 ± 732	3008 ± 599	3497 ± 1143	0.187
Ultimate stress, MPa	276 ± 35	292 ± 25	280 ± 18	280 ± 32	0.542
Modulus, GPa	11.4 ± 2.1	12.4 ± 1.9	12.3 ± 1.7	12.0 ± 2.8	0.689
Toughness, MJ/m^3^	13.1 ± 1.9	14.9 ± 2.2	12.8 ± 3.1	14.0 ± 4.1	0.312

Data presented as mean and standard deviation. p < 0.05 vs (*) VEH, (#) ALN, (&) Combo

## Discussion

There is a growing interest in the concept of combination drug treatment for treating osteoporosis [5]. Few studies have combined two anti-remodeling agents based in part on the assumption that they would both be targeting osteoclast activity. Since raloxifene has recently been shown to positively affect bone material properties via enhanced skeletal hydration [6–8], we aimed to determine if these effects would combine with those of bisphosphonates to produce superior mechanical properties than either therapy alone. Although previous work in rats supported the idea of a mechanical benefit to combining alendronate and raloxifene [31], the current study showed no benefit of combination treatment on mechanical properties, or a multitude of other skeletal properties, in a non-ovariectomized beagle dog model.

The premise of the current work was based on previous beagle dog studies, in our lab and others, showing that alendronate treatment improved bone mass and structural-level mechanical properties while also compromising tissue-level properties (mainly toughness) [14–16,19,20,32,33]. These previous studies from our laboratory utilized the same age/breed/sex of dog and the same alendronate doses as those used in the current work, yet the results have striking differences. Despite the reduction in bone remodeling being similar (current study BFR/BS was -71% vs VEH at iliac crest; previous work -67% vs VEH at vertebra [22]), there was no significant effect in the current study on properties such as vertebral trabecular BV/TV, aBMD, or any structural biomechanical properties of the rib, vertebra, femoral neck, or femoral diaphysis. One key difference with past work in our laboratory is that this study dosed animals with alendronate synthesized in-house, while previous studies used the compound provided from the drug manufacturer. The NMR spectra of the two compounds and the suppression of bone remodeling was similar, however, so it is unlikely that the drug itself explains the lack of mechanical property differences.

In the same way that alendronate failed to produce many effects seen previously, our raloxifene monotherapy group also had distinct differences compared to past data. There was no positive effect of raloxifene dosing on either structural or material-level mechanical properties as has been noted previously [22,23]. Raloxifene-treated animals had modest reductions in remodeling (current study BFR/BS was -21% vs VEH at iliac crest; previous work -18% vs VEH at vertebra [22]) and also had significantly higher percent bound water in the tibia cortex, assessed in vivo at 6 and 12 months in a subset of animals (Fig 2C). This effect on hydration is consistent with at least part of the mechanism of action of raloxifene, that of enhanced hydration of the tissue [6–8]. The reason this did not translate into alterations in whole bone or tissue-level estimates of mechanical properties in any of the bone sites assessed mechanically (rib, vertebra, femoral neck, femoral diaphysis) is not clear. In fact, rib mechanical properties were significantly lower than all other groups, although this was clearly due to the ribs being smaller as normalization for size negated all of the group differences.

Combining alendronate and raloxifene reduced remodeling to the same degree as alendronate alone and increased bone hydration (percent bound water) to the same degree as raloxifene monotherapy (Figs 1 and 2). The absence of additional suppression on remodeling beyond either monotherapy is consistent with the clinical study that combined these two agents [12]. There were no positive effects on mechanical properties that exceeded either monotherapy, a finding that is different than our hypothesis, but also inconsistent with work in ovariectomized rats [31]. In fact, the most interesting aspect of this study was the observation from the femoral neck tests where half of the combination treated group displayed distinct fracture patterns. The femoral neck test is designed to produce a bending/shear moment on the neck [34]. Of the 48 bones tested in this configuration, 6 of the bones fractured at the femoral shaft rather than the neck, and all of these were in the combination treatment group. Prior to the femoral neck mechanical tests, all specimens were scanned (at 50% length) and then underwent 3-point bending. Although the region of potting for the femoral neck tests was outside that of the support spans (and thus in theory not affected by the test), it is possible that some residual effects of the diaphysis bending test influenced the femoral neck test. This however, doesn’t change the fact that these shaft fractures were only in the combination treatment group, and occurred in 50% of the bones. The structural/tissue level effects that underlie these fracture patterns in the combination treatment group are not clear but deserve further exploration especially given the rising interest in atypical femoral fractures [2].

This study undertook in vivo assessments of bone properties using three technologies—UTE-MRI, Osteoprobe, and BioDent. UTE-MRI has the ability to measure skeletal hydration both ex vivo and in vivo [27,35–37]. We have previously reported data from the 6-month time point of these same animals and shown that raloxifene-treated animals had significantly higher percent bound water of the tibial cortex compared to vehicle-treated animals [7]. These higher values in raloxifene-treated animals were retained at the 12 month time point (Fig 2C) [37]. The positive effect of raloxifene on bound water was noted in animals treated with the combination of alendronate and raloxifene to the same degree as raloxifene monotherapy. These data suggest that positive effects on bound water are not negated by tissue-level changes induced by bisphosphonates. Recent work has shown that raloxifene can in fact normalize tissue-level properties in models where the negative effects of bisphosphonates exist [11].

BioDent and Osteoprobe are two devices developed for assessing properties of the bone tissue under conditions of cyclic and impact indentation [38,39]. There remains some controversy over various aspects of these devices [40], and what properties of the tissue they are measuring are unclear, yet we chose to incorporate them into the current study to determine if they could detect in vivo differences brought about by pharmacological therapy. Data from raloxifene-treated animals at 6 months showed that compared to vehicle-treated animals there were changes that were consistent with greater energy absorption capacity of the tissue [24]. In the current analysis, incorporating time and treatment, there was a significant effect of both alendronate and combination treatment that yielded lower total indentation distances. Based on some of the initial work with the BioDent machine this would be interpreted as a positive mechanical effect, given that indentation was shown to be inversely related to tissue toughness [38,41]. More recent work with these machines has illustrated that the relationship between indentation properties and whole bone and tissue-level mechanics is not straightforward [42]. The Osteoprobe device was not able to separate the groups and only showed a significant effect of time with lower values at 12 compared to 6 months when all animals were pooled. Based on the literature this would suggest all animals lost mechanical competence over time [39,43] although direct relationships between BMSi and mechanics are tenuous.

Beyond those aspects of the study outlined above, the work has various limitations. The animals in the study are not estrogen-deficient or osteoporotic, for reasons routinely described in our previous work using this model. Given that dogs have naturally low estrogen levels, ovariectomy tends not to mimic high-turnover conditions as occurs in other species including humans. An advantage of the dog model, as opposed to rodents, is the presence of intracortical remodeling which presents a physiological system of cortical bone similar to humans. The group sizes of the study (12/gp) were based on previous work based on mechanical differences in quasistatic tests; higher numbers may be necessary for some of the in vivo measures. Most of all, though, the work is limited by the lack of the expected effects of the two monotherapies on bone morphology/BMD and mechanical properties (such as toughness), making definitive conclusions about the effectiveness of combination treatment difficult.

In conclusion, we present a suite of data in a beagle dog model treated for one year with clinically-relevant doses of alendronate and raloxifene monotherapies or combination treatment with both agents. Despite the expected effects on bone remodeling, our study did not find the expected changes in bone geometry/architecture of the two monotherapies, and thus the viability of the combination therapy remains unclear.
